# Linking the global positioning system (GPS) to a personal digital assistant (PDA) to support tuberculosis control in South Africa: a pilot study

**DOI:** 10.1186/1476-072X-5-34

**Published:** 2006-08-16

**Authors:** Barry Dwolatzky, Estelle Trengove, Helen Struthers, James A McIntyre, Neil A Martinson

**Affiliations:** 1 School of Electrical and Information Engineering, University of the Witwatersrand, PO Box 542 Wits 2050 Johannesburg, South Africa; 2 Perinatal HIV Research Unit, University of the Witwatersrand, Johannesburg, South Africa; 3 Johns Hopkins University Center for TB Research, Baltimore, MD, USA

## Abstract

**Background:**

Tuberculosis (TB) is the leading clinical manifestation of HIV infection and caseloads continue to increase in high HIV prevalence settings. TB treatment is prolonged and treatment interruption has serious individual and public health consequences. We assessed the feasibility of using a handheld computing device programmed with customised software and linked to a GPS receiver, to assist TB control programmes to trace patients who interrupt treatment in areas without useful street maps. In this proof of concept study, we compared the time taken to re-find a home comparing given residential addresses with a customised personalised digital assistant linked to a global positioning system (PDA/GPS) device. Additionally, we assessed the feasibility of using aerial photographs to locate homes.

**Results:**

The study took place in two communities in Greater Johannesburg, South Africa: Wheillers Farm, a relatively sparsely populated informal settlement, and a portion of Alexandra, an urban township with densely populated informal settlements. Ten participants in each community were asked to locate their homes on aerial photographs. Nine from Wheillers Farm and six from Alexandra were able to identify their homes. The total time taken by a research assistant, unfamiliar with the area, to locate 10 homes in each community using the given addresses was compared with the total time taken by a community volunteer with half an hour of training to locate the same homes using the device. Time taken to locate the ten households was reduced by 20% and 50% in each community respectively using the PDA/GPS device.

**Conclusion:**

In this pilot study we show that it is feasible to use a simple PDA/GPS device to locate the homes of patients. We found that in densely populated informal settlements, GPS technology is more accurate than aerial photos in identifying homes and more efficient than addresses provided by participants. Research assessing issues of, confidentiality and cost effectiveness would have to be undertaken before implementing PDA/GPS – based technology for this application. However, this PDA/GPS device could be used to reduce part of the burden on TB control programs.

## Background

"Globally there were 4.4 million new cases of tuberculosis in 2003 [1] of which a quarter were in Africa where the HIV epidemic has resulted in rapid increases in TB caseloads. HIV infection is the greatest risk factor for TB disease and current TB control strategies appear insufficient to halt the swift rise in new cases of TB consequent to HIV [2,3]. Curative therapy for TB is complex and lasts for six to eight months. Lack of knowledge of the disease, rapid subjective responses to TB treatment, travel barriers, stigma, adverse effects of medication and poor user experiences with an overburdened TB control programme (TBCP) may result in patients not completing their full course of treatment [4-7]. Maintaining patients on TB treatment is critical in ensuring cure. Interrupted or intermittent TB treatment may result in the development of resistance to commonly used TB drugs, worsening clinical condition and, particularly in HIV-infected individuals, death.

To measure the successful implementation of the World Health Organisation's directly observed treatment, short-course (DOTS) strategy [8], a target of at least 85% of patients started on TB treatment should complete their full course of therapy. Achieving this requires that ≤ 15% of patients started on TB treatment have one of five other outcomes. The outcome that best reflects the inefficiency of the TBCP is the treatment-interrupted category. In South Africa, which ranks third by treatment interrupter rates, the cure rate is 54% and treatment interrupter rate 13% [1].

A contributing factor to high treatment interruption rates is the limited capacity of TB clinics to trace patients who interrupt treatment, a problem compounded by topography and inadequate addresses [9]. Several studies have highlighted the problems of retaining cohorts of adults started on TB treatment. For example, of a large cohort of cases referred from hospital to surrounding clinics for continuation of TB treatment in Soweto, South Africa, 21% did not attend their local clinic and 57% of those who did not attend could not be traced at their address [10]. Similarly, in Madagascar, of 95 TB patients who interrupted their treatment, 57 could not be traced. Of these, 34 had incomplete or incorrect addresses recorded [11]. A quality improvement report on the TBCP in Mzuzu District, Malawi identified incorrect addresses as being a major problem contributing to low case retention [12]. In an attempt to obtain correct addresses, two novel address card responses were tried in Tamil Nadu, India [13,14].

In many South African urban areas, a four to five digit property number followed by the community name identifies residences. Thus a single digit address error could have major implications for locating a residence. Additionally, many informal settlements have not been surveyed and do not have property numbers, and in urban settings three to six one-roomed backyard shacks have been erected on the same property as formal housing [15,16]. Because an accurate address is required to trace TB patients who do not return for continuation of their TB treatment, any intervention that could assist in locating residences of defaulting patients and which could contribute to reductions in treatment interruption is urgently needed. Both GPS and geographical information systems (GIS) have been used extensively to assess the spatial epidemiology of malaria, tuberculosis and other infectious diseases Also, these technologies have been used to evaluate the distribution of tuberculosis health facilities and lay health workers [26, 27, 28]. The objective of this pilot research project was to investigate the feasibility of using a Global Positioning System (GPS) receiver linked to handheld computing technology (such as personal digital assistants or PDAs) to enhance the tracing of patients within the TB control program.

## Results

The study took place in October and November 2005 when participants were recruited at two clinics.

### Locating homes using aerial photographs

In Wheillers Farm, nine of the 10 participants were able to locate their homes correctly on an aerial photograph. However, in the densely populated Beirut section of Alexandra with many shacks on each demarcated property, only six out of ten were able to locate their homes.

### Comparing given addresses to the PDA/GPS device

In both communities, a research assistant unfamiliar with the community, was able to locate nine out of ten of the participant's homes having been given their name and street address. However problems were encountered in finding virtually all addresses provided. Names of streets were not clearly marked or were not in common usage and/or house numbers did not follow logical patterns. In one instance shacks that had been relocated due to installation of infrastructure retained their original stand numbers despite being moved to a new location. In Alexandra, a single street address was found to comprise 10 separate households.

In both areas a volunteer recruited at the clinic who was previously unfamiliar with GPS devices, located the same 10 homes without assistance using the programmed PDA. Table 1 shows the total time taken to find the homes of the clinic participants by the lay volunteers comparing time taken using given addresses with that using the PDA/GPS device.

**Table 1:** Comparison of total time taken by a lay volunteer recruited at the clinic comparing given addresses with GPS coordinates to locate a participants residence

	**Wheillers Farm (n = 10)**		**Alexandra (n = 10)**	
	Name and address	PDA/GPS	Name and address	PDA/GPS

Number found	10	10	9	10
Total time taken	5 hours	4 hours	3 hours	1 hr 30 min

## Discussion

This is the first report we are aware of that assesses the role of GPS in improving the efficiency of TB patient tracing and in which PDAs are used linked with GPS. We piloted a novel information technology approach that could potentially improve patient retention within TB control programmes. Using the specially developed PDA/GPS application, lay volunteers with minimal training were able to record the GPS coordinates of a patient's home and using the same prototype application, different lay volunteers were able to locate all of the patients' homes using their GPS coordinates even in the narrow confines of densely populated informal settlements of Alexandra.

A research assistant observed the volunteers and it appears they had no difficulty in using our prototype PDA/GPS device to locate a home. Minimally skilled workers could easily implement this process and as most clinics in South Africa use lay health workers to perform counselling and administrative functions, this category of personnel could easily operate the device. In addition, the use of lay health workers has been shown to somewhat improve the outcomes of TB control programs [29, 30]. However, time would have to be allocated for them to accompany all patients recently diagnosed with TB to their homes to record GPS coordinates.

Limitations of this study are that sample size is too small to draw statistically relevant findings from the study and communities were not randomly selected. The comparison of total time taken of the research assistant, unfamiliar with the community, to that of the lay volunteer using the PDA/GPS device may be biased as in a real situation community health workers performing a tracing function would become familiar with a community and may be able to locate homes more efficiently than someone without prior knowledge of the community. Furthermore, like other GPS applications where residences are located and entered into a database, patient confidentiality could be compromised. However, both names and addresses are a required field in the current TB registers used in all TB clinics and some of these registers are electronically entered in South Africa. If this application was implemented on a wide scale, an alternative form of identification to names, such as the national personal identification number or a TB register number, may be more appropriate. Acceptability of this method of tracing by people who are both HIV-infected and have TB may be less compared to that of the clinic participants in our study whose HIV and TB status was not known. In a large proportion of TB patients – those who are 100% adherent – the home GPS coordinates may never be required. The effort and costs of a lay worker required to locate homes of all TB patients soon after diagnosis would have to be weighed up against the potential benefits in tracing defaulters as the home coordinates would be valuable in approximately 13% of TB patients who interrupt treatment and in the 10–25% of patients who die without the TBCP being informed. The potential benefit of aerial photographs is that they would not require an initial home visit.

The system we developed could be linked to an electronic TB register or database where cyclical queries could be generated that identified those patients not returning for continuation of their medication who would then be traced. In addition, using the home locations of TB patients, the GPS coordinates could be used to map outbreaks of TB or drug resistant TB. Other potential applications for the technology piloted here could be the tracing of study participants in clinical trials and recording household location in census and household surveys. In all applications, the PDA could be programmed for direct data entry by staff members as has been done in other poorly resourced settings [31]. Inexpensive handheld GPS receivers with back-tracing facilities e.g. the Forotrex receiver (Garmin International Inc. Olathe, KS) could be used for this application but may be more difficult to use by a layperson and would not have the data-entry applications or adaptability of the PDA/GPS device, particularly the potential to link with comprehensive health information software.

## Conclusion

We have shown preliminary evidence in this pilot study that PDA/GPS devices can easily be used to locate clinic attendees' homes successfully using unskilled lay personnel with minimal training. Aerial photographs can be used in locating homes in low dwelling-density informal settlements but are less accurate in high-density settlements. The GPS linked PDA could easily be coupled to the TB health information system using information technology to improve TB control programs that are currently struggling to cope with the burden of HIV-related TB.

## Methods

### Selection of areas and participants for the study

Two residential areas, both in the greater Johannesburg area, were selected for this study on the basis of convenience. We selected the two settings to test the relative effectiveness of using the devices in two very different urban locations, both where HIV and TB are significant public health problems. Both communities have clinics that diagnose and treat TB. The communities were: Wheillers Farm, a recently occupied informal settlement south of Johannesburg whose predominant housing type is shacks built by their occupants. Dwelling densities are two to three per demarcated property. It is laid out on a grid pattern, and has electricity and piped water. The second area is the Beirut section in Alexandra. Alexandra is a densely populated township in Johannesburg -about four by four km. Although it has a formal layout, with named streets, extensive unplanned development has occurred over many years. Shacks have been erected in the yards of houses, along watercourses and on unused land. The Beirut section is in the south western part of Alexandra and includes both formal and informal housing.

Two research assistants (final year engineering students) were employed to assist with the study. After an initial training session, they visited the clinics in Wheillers Farm and Alexandra where they recruited 10 adult attendees at each clinic after obtaining informed consent. For the purpose of this pilot study the medical condition of participants was irrelevant – the patients were not necessarily infected with TB or HIV. Participants were recruited from the waiting area of the clinic. The study was approved by the Human Research Ethics Committee (Medical) of the University of the Witwatersrand, Johannesburg.

### Using aerial photographs to locate homes

The research assistant pointed out four well-known local landmarks on an aerial photograph of the area, and then requested the participant to attempt to locate his/her home without assistance and marked this location on the photograph. Our original intention was to use street maps of the two areas to compare ability to locate households on maps with aerial photographs. However, we were unable to find street maps with sufficient detail. In Alexandra, street maps do not reflect the short side roads entering a group of houses and in Wheillers Farm, the available street maps only showed main roads without showing newer unsurfaced roads.

### Collecting address and GPS coordinates

Participants were asked to provide their residential addresses and names which were written down. The research assistant then walked from the clinic with each participant to his/her home. At the participant's home, the GPS location was immediately downloaded to the PDA from the GPS device by the research assistant using a customised software application on the PDA via Bluetooth.

### Locating participants' homes

Several days after the data collection exercise the research assistants returned to the clinics. One of the research assistants, not familiar with the area, attempted to locate the homes of the participants whose addresses had been recorded a few days earlier. Apart from the address provided by the participant, he was blinded to the location of the residence and had to rely on the address and name provided. He was allowed to ask passers-by and neighbours for directions. The research assistant did not return to the clinic after finding each residence.

A lay volunteer was recruited in each of the communities. These volunteers had never worked with PDAs or GPS. At each site, one of the research assistants gave the lay volunteer a short training session (less than 30 minutes) on the use of the PDA/GPS software application. The volunteer then used the PDA to locate the home of each participant, based only on the customised program in the PDA which directed the volunteer, by means of an arrow, to the stored GPS position. The volunteer was blinded to the names of the participants and to their street addresses. Another research assistant who had not previously been to the homes of participants accompanied the volunteer as an observer providing no assistance in the use of the device or in directing the volunteer where to go. As with the previous exercise, residences were visited sequentially without returning to the clinic and a total time to find all ten residences was recorded.

### Brief description of the prototype PDA software application

**Figure 1:**
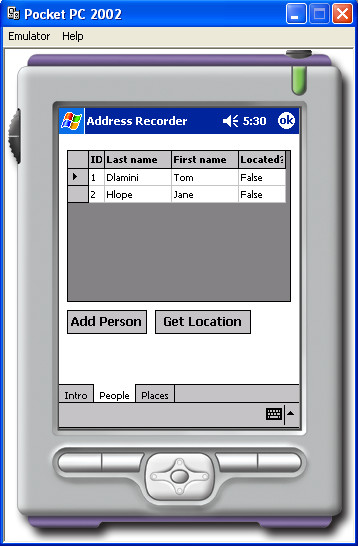
The interface used for recording the patients' names and GPS coordinates.

**Figure 2:**
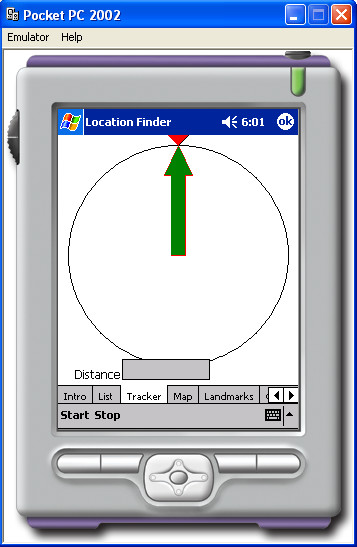
The "Location Finder" application features a simple arrow that directs the user to the target GPS position.

A Hewlett Packard (Palo Alto, CA) iPAQ 8000 PDA, with Microsoft (Redmond, WA) Pocket PC 2002 as its operating system was used in this study. A separate GPS receiver (Fortuna Bluetooth) was linked to the PDA using a short-range wireless connection: Bluetooth (Bellevue WA). The total cost of the hardware was USD730. Customised software was developed for the PDA by an experienced programmer, written in C# programming language, using the Microsoft.NET framework. This configuration was selected because of the flexibility it offered in developing our prototype. By keeping the PDA and GPS receiver separate a number of different combinations of devices could be tested. In addition, the PDA provides a development platform that can be easily programmed using Visual Studio.Net and a combination of C#.Net and Visual Basic.Net. The GPS.Net Global Positioning SDK (StormSource Software LLC, Lakewood, CO) was used in the development of our application. Approximately 30 hours of programming were required to develop and de-bug two separate PDA applications: "AddressRecorder", for collecting GPS coordinates of participants' homes and "LocationFinder", used to direct a pedestrian user with an arrow to the participant's home, given its GPS coordinates. The GPS coordinates were downloaded into an XML file. In this format they can easily and conveniently be exported into a GIS. No maps were used by the program. The key requirement for both applications was ease of use by people with no previous experience in using either PDA or GPS devices or computers. Screen dumps (Figures 1 and 2) demonstrate interfaces developed for this study.

## Competing interests

All equipment and software used were purchased from the suppliers. No equipment was donated and no sponsorship from commercial vendors was received by any of the authors.

## Authors' contributions

BD, ET, HS and NM formulated the idea for this research. BD designed and developed the prototype software used in the study, wrote the results, methods and background sections of this paper. ET managed the research assistants and procured aerial photographs. NM facilitated clinic access, wrote the background and conclusions sections and edited the paper. HS and JM assisted with the original grant application, and overall management. All authors read and approved the final manuscript.
